# Handling the desire to die– evaluation of an elective course for medical students

**DOI:** 10.1186/s12909-024-05269-6

**Published:** 2024-03-18

**Authors:** M Schallenburger, J Schwartz, Yann-Nicolas Batzler, St Meier, R Küppers, Th Tenge, A Doll, K Kremeike, D Wetzchewald, M Neukirchen

**Affiliations:** 1 Interdisciplinary Centre for Palliative Medicine, Heinrich-Heine-University Duesseldorf, University Hospital Duesseldorf, Duesseldorf, Germany; 2 Centre of Integrated Oncology Aachen Bonn Cologne Duesseldorf (CIO ABCD), Cologne, Germany; 3 Department of Anaesthesiology, Heinrich-Heine-University Duesseldorf, University Hospital Duesseldorf, Duesseldorf, Germany; 4 Centre for Palliative Medicine, University Cologne, University Hospital Cologne, Cologne, Germany; 5 Institute for Emergency Medicine, University Witten/Herdecke, Arnsberg, Germany

**Keywords:** Desire to die, Death wishes, Medical education, Assisted dying, Undergraduate medical studies, Attitude towards assisted dying, Voluntary stop eating and drinking, Assisted suicide

## Abstract

**Background:**

The desire to die can occur in palliative care patients with a prevalence of up to 22%. Not every desire to die is accompanied by a pressure to act, but usually by a burden that can arise from various factors. To address this burden appropriately, health care workers should be trained. Based on an evaluated course on handling the desire to die, an elective course for medical students was developed and evaluated. In order to identify the impact of the elective course’s content, a comparison of attitudes towards assisted dying with two other participant groups was conducted. Therefore, three questions from the evaluation of the elective course were used.

**Method:**

Online evaluation of the elective and questions addressing attitude were assessed using a five-point Likert scale. The specific outcome-based assessment was determined using the Comparative Self-Assessment Gain. The main participant group (group 1) were students who took the elective. The additional survey on attitudes towards assisted dying included undergraduate medical students who had taken compulsory palliative care courses (group 2) and physicians who had taken an introductory course in intensive care or emergency medicine (group 3).

**Results:**

Group 1 (*n* = 13, response rate rr = 86.7%) was very satisfied with the blended learning format (100%) and the course itself (100%). They were able to deepen their knowledge (81.0%) and train skills (71.2%) through the course. In the additional surveys, there were 37 students in group 2 (rr = 66.1%) and 258 physicians in group 3 (rr = 73.6%). Willingness to assist with or accompany the various options for assisted dying varied according to the type of assistance. Among the participants, it can be summarised that the highest willingness was shown by the students of group 2 followed by the physicians of group 3 and the students of group 1.

**Conclusions:**

A course on handling the desire to die of palliative patients can deepen knowledge and train communication skills and thus support self-confidence. Dealing with the background of the desire to die, knowledge about assisted dying, but also one’s own attitudes and responsibilities can influence the attitude towards assisted dying.

**Supplementary Information:**

The online version contains supplementary material available at 10.1186/s12909-024-05269-6.

## Background

The desire to die describes a phenomenon that occurs primarily in individuals with life-limiting progressive illnesses. The desire to die is described as a continuum ranging from acceptance of death to acute (consciously planned) suicidality. Between these two poles, the pressure to put the wish into action increases in hopes of dying soon or in the wish to hasten death [1].

The wish to hasten death, which the study focuses on, is defined as a reaction to suffering in the context of a life-threatening illness, in which the patient sees no other option than an accelerated dying. It is a manifestation of desire to die with a medium pressure to act. One or more physical, psychological, social or spiritual factors or symptoms can lead to such a reaction [1]. It can thus be differentiated from suicidal thoughts of people without a life-limiting illness.

In patients with non-curable cancer, the prevalence of a desire to die ranges from 8 to 22% [2, 3]. It is important to mention that this wish can also arise and be expressed without any pressure to act. Not every patient with a desire to die actually wants to end their life prematurely or is suicidal. To properly care for patients and sufficiently control symptoms on all four dimensions mentioned, it is important to recognize the patients’ desire for a hastened death, to talk about it and to understand the background, such as reasons or functions.

Frerich et al. (2020) give the recommendation to actively address the presence of a wish to hasten death. However, this can be difficult for health care professionals. To strengthen them in this respect, a communication course was developed and validated [4, 5].

In order to properly prepare medical students for this delicate topic during their medical undergraduate program, an elective course in a blended learning format was developed based on the validated course. The students are guided through the topic through a specially developed case vignette. The structure can be seen in Box 1. The content is designed to teach predefined learning objectives (Table 1) at the levels of knowledge, skills and attitude. The elective is limited to a small group of students per semester due to the sensitive topic, exercises based on case simulations and an abundance of self-reflection. In 30 semester hours, divided into 24 h of face-to-face or online lectures and six hours of self-study with eLearning over 15 semester weeks, students learn about the case of a mother suffering from amyotrophic lateral sclerosis. Due to the progression of the disease and worries about being a burden on the family as well as fear of shortness of breath, she expresses the desire to end her life prematurely. The students receive theoretical knowledge on the subject in advance and learn about an interview guide, which they can put into practice within smaller case vignettes. The SPIKES [6] model could provide support and the students learn about it in the compulsory courses in palliative care. It is not used in the elective because it was developed for the purpose of breaking bad news. The focus in the elective is put on openly but specifically addressing death wishes. The sample patients are therefore already informed about their situation. The guide used here contains open-ended introductory questions related to fear of dying/death or burdens of the patients. It also offers options for categorising death wishes in terms of functions, meanings and causes [7].

The conversation simulation, in which an actress plays the patient, offers all students the opportunity to train and test individual skills. Students should recognize the covertly expressed wish to hasten death and talk about it with the patient. For self-reflection, the students are guided at the beginning of the elective course, as well as at the end in relation to the topic of self-protection. They get the opportunity to self-reflect, exchange experiences within the group setting and reflect while completing the eLearning. In addition, video sequences in which a relative of a real patient tells how she experienced her father’s voluntary stopping of eating and drinking are watched together in the face-to-face sessions Box a.

**Fig. a:**
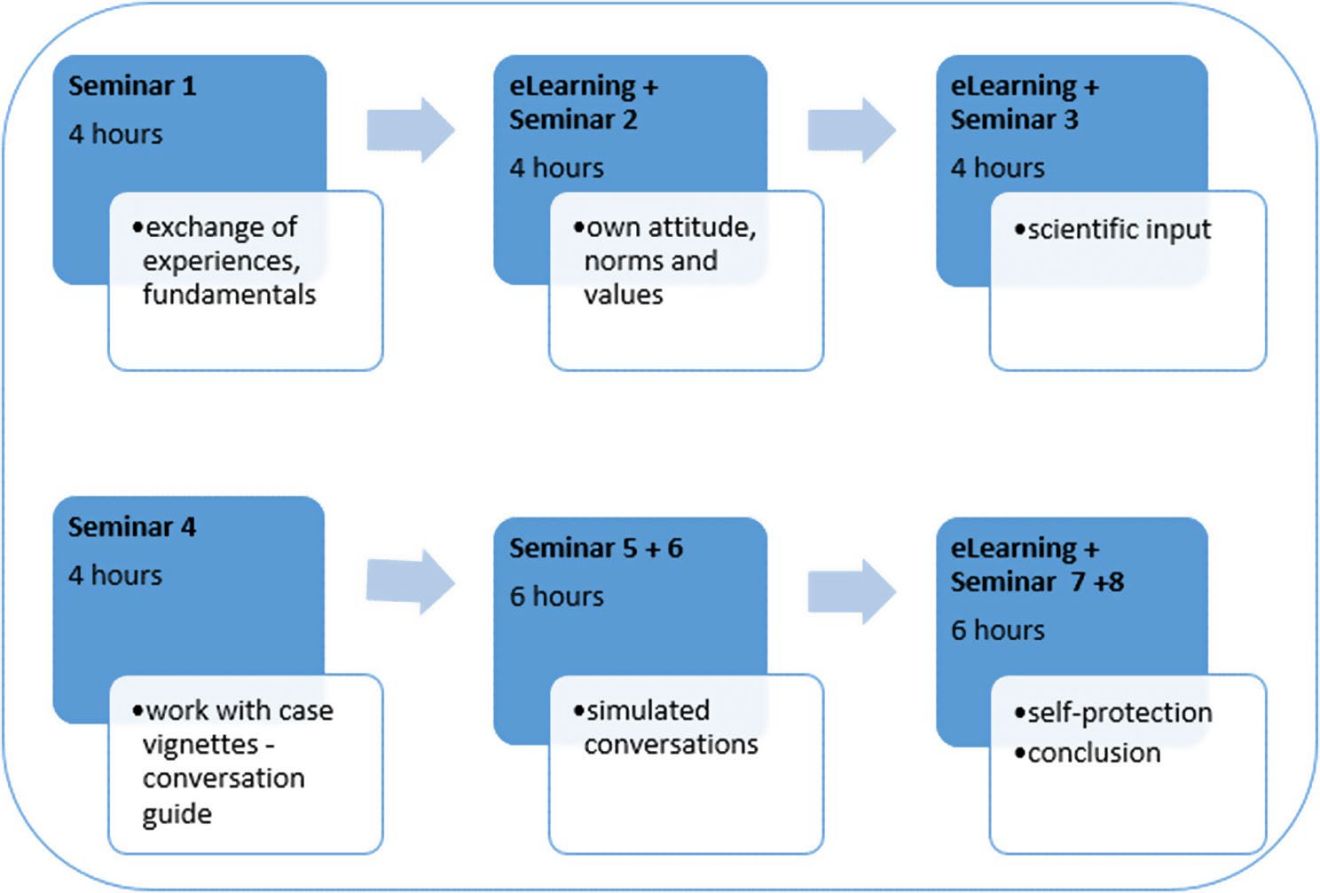
Structure of the elective course

**Table 1:** Learning objectives of the elective course

knowledge	The participants… • know about the complexity of desire to die • know about the background like reasons or suffering, that can lie behind such thoughts in affected patients • know how to react to such wishes and about possible interventions • know the different types of assisted dying • are able to evaluate different expressions of a desire to die and relate them to the background of the situation • know about the importance of involving relatives • know the current scientific perspective on the phenomenon of the desire to die • know possible symptoms of the final phase
skills	• recognise a possible desire to die in patients’ statements • can react to the expression of the wishes • can talk to patients about their desire to die • are able to start a conversation about possible desire to die • can interpret and understand patients’ fears better • are able to protect themselves
attitude	• are aware of their own values and norms in relation to dealing with a desire to die • are aware that their own values and norms can have an influence on their own attitude and thus on how they handle a desire to die • are able to adopt an open attitude towards handling a desire to die • are able to perceive their own strengths and weaknesses in handling a desire to die • are capable of self-reflection

The comprehensive content of the course is intended to give students the opportunity not only to reflect on and strengthen their attitude towards the subject. In addition, they should learn that the wish to hasten death often is not an actual wish for assisted suicide, but that there are causes, functions and meanings behind it that need to be explored. Palliative approaches, which the students learn about, question and discuss in the course, can help to support seriously ill patients and relieve their burdens that might lead to such wishes.

This study aims to determine to what extent an elective course in a blended learning format can give medical students an understanding of the topic of dealing with the wish to hasten death (aim 1). In addition, attitudes towards assisted dying were to be compared with other cohorts (aim 2).

## Method

An evaluation of the elective course was conducted consisting of 15 items (see, Table 3). To assess attitudes on voluntarily stopping of eating and drinking (VSED), assisted suicide and death by request, three items (XVI to XVIII, Table 3) were added. Besides students taking the elective, these three items were answered by medical students attending compulsory seminars in palliative care as well as young physicians attending an emergency or intensive medicine course. This allowed for a comparison of different cohorts in regards of views towards assisted dying.

### Participants and procedure

The elective course can be chosen throughout the clinical part of the medical curriculum at our faculty. Compulsory courses in palliative medicine take place in the final year of study, therefore most of the students that choose the elective have not yet received a course in palliative medicine beforehand. All participants gave their informed consent to take part in the study. The study was approved by the ethics committee of the Heinrich Heine University Duesseldorf (Study-ID: 2021 − 1592).

All students who took the elective course on dealing with a desire to die were invited to participate voluntarily in this evaluation study (group 1). The evaluation comprised both a global teaching evaluation and a specific outcome-based assessment. The global evaluation took place after completion of the elective. The specific outcome-based assessment was recorded using the post-then method (i.e. retrospectively). Data was collected electronically using Unipark® software (Manufacturer Tivian XI GmbH).

Within the evaluation questionnaire, three items (XVI to XVIII, Table 3) covered attitudes towards different ways of assisted dying. Besides the students who took the elective (group 1), these items were answered by different cohorts in two further surveys. The groups are presented separately in Table 2. The participants were, on the one hand, medical students who had completed compulsory courses in palliative medicine (group 2). They receive these in their fifth year of study. On the other hand, physicians who had participated in an introductory course in intensive care medicine or a seminar in emergency medicine (group 3) were asked to answer the items. These are residents in the first years of their specialty training. The survey also took place using Unipark® software.

**Table 2:** Description of the participant groups

name	group 1	group 2	group 3
**participants**	participants of the described elective main cohort	medical students who had completed compulsatory courses in palliative medicine comparison cohort	physicians of an introductory course in intensive care medicine or a seminar in emergency medicine comparison cohort
**evaluation**	global evaluation and specific outcome-based assessment	Items XVI to XVIII of the global evaluation	Items XVI to XVIII of the global evaluation
**timing of evaluation**	after completion of the elective course	after completion of compulsory courses in palliative care	no specific point in time; during the course

### Measures

The structured questionnaire developed for the evaluation of the elective course comprised 31 items (Tables 3 and 4) including the three that were used for the supplementary surveys (Table 3 Questions XVI– XVIII). It was developed specifically for this study by the study team in German language. It consists of two parts, the global evaluation (Table 3) and the specific outcome-based assessment (Table 4). The learning objectives of the compulsory courses of palliative care in undergraduate medical school and of the elective served as criteria for the development of the questionnaire. They take into account the ten core competencies of the European Association of Palliative Care (EAPC) [8] and are oriented towards the German Qualifications Framework (DQR) for lifelong learning [9]. According to the DQR, learning objectives must reflect both professional competences (i.e. knowledge and skills) and personal competences (i.e. social competence and autonomy). The latter describes the ability and willingness to personally develop and adapt independently and responsibly in the respective social, cultural or professional context and can be understood as an attitude. Professional and personal skills are understood as so-called action competences according to the DQR.

**Table 3:** Items of the global evaluation of the elective course

Question	Subject	Wording
I	demographics	Gender
II	demographics	Age
III	prior experience	Do you have any previous experience in a health care profession besides the medical undergraduate programme and the included practicums?
IV	prior experience	Have you had any experiences in your professional life with death wishes expressed by patients?
V	didactics	The didactic structure of the elective is well done.
VI	structure	The structure of the elective in eLearning, self-study and (web) seminar is successful.
VII	eLearning	The contents of the eLearning are presented in an understandable way.
VIII	didactics	The lecturers appear competent.
IX	didactics	The accompanying (web) seminars make digitally conveyed content more understandable.
X	didactics	The digital implementation of the web seminars is equivalent to a real encounter.
XI	structure	The time frame for the exchange among each other is appropriate.
XII	didactics	The content of the elective prepares well for the simulated conversations.
XIII	self-competence	Through the simulated conversations, I have gained confidence in handling expressed desire to die.
XIV	attitude	The elective has changed my attitude towards desire to die.
XV	Overall satisfaction	Overall, I am satisfied with the elective course.
XVI	attitude VSED	I can imagine support a patient during the VSED.
XVII	attitude assisted suicide	I can imagine offering physician assisted suicide to a patient.
XVIII	attitude death on request	I can imagine carrying out death by request (forbidden in Germany).

**Table 4:** Items of the outcome-based assessment

Question	Wording	Competence
1	I believe that dealing with patients’ desire to die is an important issue in my future professional work.	attitude
2	I am aware of my own attitude to the issue of desire to die.	attitude
3	I can reflect on the influence of norms and values on my attitude towards the desire to die.	skills
4	I have the confidence to proactively address a desire to die with patients.	skills
5	I can perceive and recognise a desire to die expressed by patients.	skills
6	I experience expressions of a desire to die by patients as burdensome.	attitude
7	I recognise the different functions of a desire to die.	knowledge
8	I know about the meaning and reasons of a desire to die.	knowledge
9	I know the legal basis of assisted dying in Germany.	knowledge
10	I am able to define the different forms of assisted dying.	knowledge
11	I know my resilience resources.	skills
12	I know symptoms that belong to the final phase of dying.	knowledge
13	I trust myself to care for a patient with a desire to die.	skills

The global evaluation with 18 items (see Table 3) included demographic information (Items I and II) as well as previous experiences in a health profession and dealing with a desire to die expressed by patients (Item III and IV). Items V to XV measure the structure and didactics of the elective. Items XVI to XVIII cover attitudes towards VSED, (physician-) assisted suicide and death by request (forbidden in Germany). Participants rated the items on a five-point Likert scale from “strongly agree” to “strongly disagree”, with “strongly agree” and “somewhat agree” as affirmative, i.e. positive, answers.

The outcome-based assessment of the elective comprised 13 items (see Table 4). This specific outcome is based on a comparative self-assessment (CSA). In a retrospective post-then evaluation, students assessed the achievement of specific learning outcomes in the areas of knowledge (items 7,8,9,10,12), skills (items 3,4,5,11,13) and attitude (items 1,2,6). The possible response options were evaluated according to the German school grading system (1 = very good to 6 = insufficient).

The supplementary surveys included only items XVI to XVIII on attitudes towards VSED, (physician-) assisted suicide and death on request (prohibited in Germany).

### Data analysis

The global outcome was analysed descriptively with frequencies in percent.

The specific outcome-based assessment by means of CSA was calculated using the following formula [10]:

$$ CSA Gain \left[\%\right]=\frac{\text{m}\text{v}\text{p}\text{r}\text{e}-\text{m}\text{v}\text{p}\text{o}\text{s}\text{t}}{\text{m}\text{v}\text{p}\text{r}\text{e}-1}\times 100$$

mv_pre_ = mean self-assessment before starting the elective.

mv_post_ = mean self-assessment after completing the elective.

## Results

### Descriptive statistics

From April 2021 to July 2022, a total of 16 students participated in the elective course in three semesters and thus three runs. 13 of them participated in the online evaluation (group 1) (response rate rr: 81.3%). The other three did not consciously refuse to participate but did not evaluate despite reminders. All participants completed the questionnaire in full, so there was no missing data.

Table 5 shows the demographics of the participants in the evaluation.

**Table 5:** Demographic data (n=13)

Demographic data (*n* = 13)		n (%)	
Gender	female		9 (69.2%)
	male		4 (37.8%)
Age	20–24 years		7 (53.8%)
	25–29 years		3 (23.1%)
	30–34 years		3 (23.1%)
previous experience in healthcare	yes		5 (38.5%)
	no		8 (61.5%)

### Global evaluation (group 1)

All students that participated in the evaluation (group 1) were satisfied with the didactic structure of the elective (100%) and the competence of the teachers (100%). The structure of the elective was rated successful (100%) and the content taught was perceived comprehensible (100%). The offer of simulation conversations also gave confidence (100%) and students felt well prepared for them (100%).

76.9% stated that in general their attitude towards the subject “the desire to die” had changed as a result of the elective. However, only 61.5% agreed that online seminars are equivalent to a real meeting.

### Evaluation of the attitude towards assisted dying (group 1–3)

**Fig. 1:**
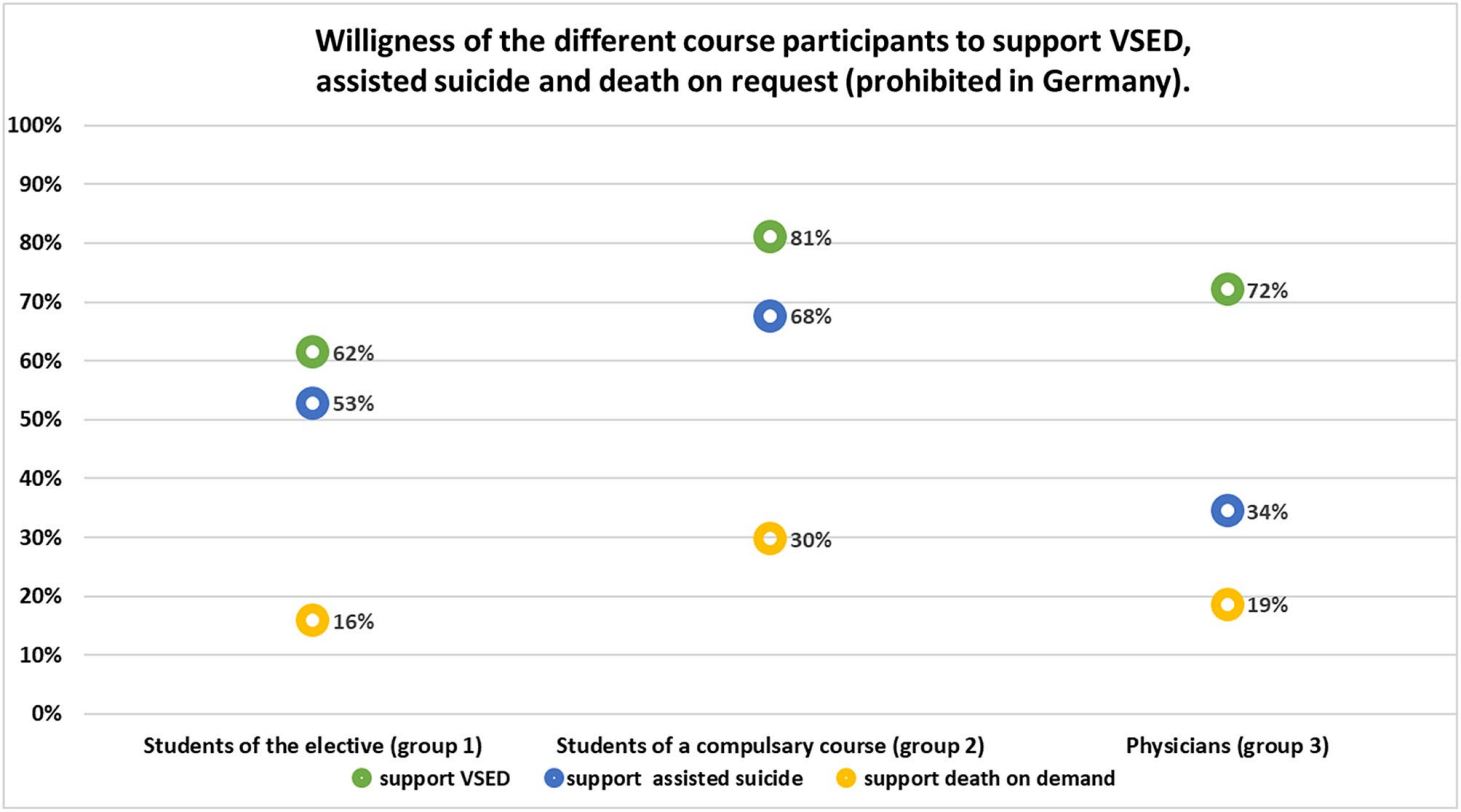
Evaluation of agreement of support for different death wishes

The rates of approval of VSED, (physician-) assisted suicide and death by request among the different groups surveyed are shown in Fig. 1. The students of the Palliative Care compulsory course (group 2) (*n* = 37, rr: 66.1%) show the highest willingness to support this form of assisted dying.

In addition to agreement, there were also refusals and abstentions (neither nor) in the three questions on willingness to support in the various assisted dying. Of the 13 participants in the elective course, 23.1% abstained from accompanying a VSED, 38.5% abstained from assisted suicide and 30.8% abstained from death by request.

In the courses surveyed for comparison, the students of the compulsory seminar in Palliative Care abstained from VSED with 5.4%, assisted suicide with 8. 1% and death by request with 24. 3%.

Out of the physicians who participated in intensive care or emergency medical training (*n* = 285, rr: 73.6%), 7% abstained from supporting a VSED, 6.3% for assisted suicide and 12.3% for death by request.

### Outcome evaluation (group 1)

In the outcome-based evaluation of the elective, the highest learning gains were achieved in the domain of knowledge (81.0%), followed by skills (71.2%) and attitude (53.5%). For example, students increased their knowledge of the different definitions of assisted dying by 89.3%. Practising to proactively address patients’ desires to die in conversational simulations improved this skill by 82.2%. Regarding their own attitude towards the subject of death wishes, 69.1% stated that they were now more aware of these wishes. For further details see Table 6.

**Table 6:** Comparative Self-Assessment (CSA) Gains of each Item

Domain	Item	CSA Gain
knowledge (total 81.0%)	7	85.1%
	8	80.0%
	9	82.2%
	10	89.3%
	12	68.5%
skill (total 71.2%)	3	72.7%
	4	82.2%
	5	77.5%
	11	63.1%
	13	60.3%
attitude (total 53.5%)	1	77.9%
	2	69.1%
	6	13.0%

## Discussion

The present study is an outcome evaluation that measures a general teaching evaluation of the elective course “Handling the desire to die” as well as specific self-assessed learning outcomes. The elective helps students to broaden their knowledge, train their skills and reflect on their personal attitudes.

Overall, the concept of blended learning is evaluated positively by the students (group 1). They perceive the opportunities for discussion with one another and the lecturers while deepening theoretical content on the topic of palliative patients’ desire to die as helpful. Adding up, they gain confidence mainly by talking to simulation patients. Furthermore, making use of eLearning showed great success in delivering professional skills as confirmed by literature [11–13].

We also found, like in other evaluated palliative care courses [14], that the highest learning gain is achieved at the level of knowledge and the lowest at the level of attitude. In this study, however, it is still quite high at 53.5%. As mentioned before, personal competences describe the ability and willingness to develop and adapt independently and responsibly in the respective social, cultural or professional context, which can be understood as an attitude [9]. The learning gain on the level of attitude can therefore be attributed to the contents of the elective and here especially to the support of self-reflection, exercises with simulation patients, but also to the integration of video sequences with real relatives.

By engaging with the contents of the elective course, the students (group 1) were able to gain confidence in dealing with patients with a desire to die. In palliative care patients, active management of such desires is recommended to show patients that they can talk openly about them [15]. This also strengthens the physician-patient relationship, which is essential for such conversations. There should be no concern that actively engaging with wished to die might trigger suicidal wishes or thoughts. If such thoughts are not present in the contacted patients, they will not be triggered by an active approach [16]. Through the conscious imparting of knowledge and the implementation in practical exercises, the participants not only dare to recognise the desire to hasten death, but also to openly address and question it. This shows that conversation simulations with simulation patients can create self-confidence. It therefore makes sense to give this method more time so that students can try out different situations and at different times. In addition to repetition, simulated patients offer the advantage that real patients do not need to be involved and experience additional stress, however, the students still experience a reasonably real situation. Direct feedback from the simulation patients can increase the learning effect [17].

The students (group 1) deepen their knowledge of the different backgrounds that can lead to the desire for a hastened death. Likewise, possible approaches to symptom reduction on different levels are learned. The experience of relatives and palliative physicians shows that the desire for a hastened death that arises at the beginning of an illness can often be reduced by talking about the possibilities of symptom relief and alternatives such as palliative sedation in the case of severe and untreatable symptoms [18]. The content of the elective, which is aligned with this, thus provide students with knowledge for their future professional activities. This includes, among other things, communicative skills, knowledge about assisted dying and the support of seriously ill people with a desire to die.

An important point is to deal with one’s own attitude towards the topic of desires to die. This can help to conduct conversations appropriately [19]. Reflecting on one’s own experiences, values and norms is part of the elective described, which can have an impact on the gain in self-confidence reported by the participants. The conversation guide that is presented and used in the elective can also increase self-confidence [19].

Recognising one’s own limits and possible overload is necessary in order to be able to react empathetically to challenging situations while protecting oneself. Measures such as supervision or case conferences are recommended [15], but personal development is also necessary here. Consequently, an attempt should be made to allocate more time to this topic in the elective subject and perhaps carry out exercises with the support of a psycho-oncologist in the future.

When asked about attitudes towards VSED, assisted suicide and death by request, medical students in compulsory palliative care courses (group 2) indicated varying degrees of willingness to accompany VSED, assisted suicide and death by request. The physicians in the introductory courses in intensive care and emergency medicine (group 3) show a high level of willingness to support VSED, less so for death by request and a low to medium level of willingness to assist in suicide. Medical students in the elective (group 1) described above, indicate a lower willingness to participate in VSED and death by request and a higher willingness to participate in assisted suicide. These differences can be attributed both to the way the topic was dealt within the courses prior to the survey and to different responsibilities for such measures.

Participants in the elective (group 1) spend an entire semester dealing with the desire to die. During this time, they deal with the background and legal requirements, but also with their own attitudes and experiences. Physicians in the introductory courses (group 3) and students in the compulsory subject (group 2) do not deal with the topic of the wish to die to the same extent as participants in the elective course. They therefore do not receive as much detailed knowledge on these topics. Participants in the elective (group 1) learn, for example, about the emotions of a relative when supporting next of kin during VSED. The relative reports about side effects and the stress experienced. This might have influenced the answer to the question about accompaniment during VSED (Table 3 item XVI). The lower willingness of physicians (group 3) compared to students in the compulsory subject (group 2) could be due to the greater practical experience or the awareness that they not only accompany but are also directly involved and responsible. This is shown by a survey among members of the German Society for Palliative Medicine. In this survey, non-physician professional groups show a higher willingness to consent to assisted suicide than physicians who become personally involved for an assisted suicide by prescribing medication themselves [18]. In another survey, the willingness to participate among physicians decreased with increased level of training [20].

The attitudes towards the different forms of assisted dying assessed in the questionnaire are a snapshot at the time of the survey. However, attitudes and opinions can change on such a sensitive topic. This is shown, for example, by a survey among members of the German Society for Palliative Medicine. Here, attitudes are currently tending to change in the direction of advocating assisted suicide [15]. The extent to which previous experience can influence attitudes and thus potentially decisions is to be explored in greater depth in the elective subject in future.

The low number of participants in the elective subject can be named as a limitation. In addition, the different groups surveyed have different previous experience with regard to the topic of the desire to die.

The aim was to evaluate the elective (group 1), and the high response rate made it possible to obtain information on this. With regard to the questions about support for assisted dying in the two other cohorts (group 2 and 3), at least initial impressions could be gained, which should be deepened in further studies.

In the elective subject, the aim is to train how to deal with death wishes expressed by seriously ill patients. Students should be able to recognise such wishes and work out causes and relief-strategies for those affected. Assisted suicide is a topic in the elective to inform students about current regulations, but it is not dealt with in detail. However, as this topic is becoming more relevant in society, the elective subject should be adapted accordingly in the future.

## Conclusion

An open and respectful approach to patients’ expressed desires to die enables care to be tailored to individual needs. Physicians should be sensitised and trained for such conversations. The elective course presented trains skills, deepens knowledge and thus provides self-confidence for the topic by practicing knowledge about the background of desires to die and properly communicating with patients about it.

Dealing with the topic of the wish to hasten death, but also with assisted dying, offers the opportunity for reflection and thus the development of one’s own attitude, which is based on sound knowledge and experience in addition to personal approaches. Patients’ desires to die should be given time and space, together with the feelings and burden behind them.

### Electronic supplementary material

Below is the link to the electronic supplementary material.

Supplementary Material 1

Supplementary Material 2

## Data Availability

The datasets used and analysed during the current study are available from the corresponding author on reasonable request. The case vignette mentioned in the background can be found in a version translated into English in the supplementary files.

## Abbreviations

EAPC: European Association of Palliative Care
DQR: German Qualification Framework
CSA: Gain Comparative Self-Assessment Gain
VSED: voluntary stop eating and drinking
Rr: response rate
