# Supplementary material for: Handling the desire to die– evaluation of an elective course for medical students
Source: BMC Med Educ. 2024 Mar 18;24:279. doi: 10.1186/s12909-024-05269-6 (PMC10946106; doi:10.1186/s12909-024-05269-6)
Supplement: Supplementary file 1 — Supplementary Material 1 [file 12909_2024_5269_MOESM1_ESM.pdf]

Evaluation of the elective course „Handling the desire to die"

1. Please indicate your gender:
   - o female
   - o male
   - o diverse

2. How old are you
   - o 20 to 24 years
   - o 25 to 29 years
   - o 30 to 34 years
   - o 35 to 39 years
   - o > 40 years

3. Do you have any previous experience in a health care profession besides the medical undergraduate programme and the included practicums?
   - o no
   - o yes if so, how many months or years including education?
     - ◊ …… months
     - ◊ …… years

4. Have you had any experiences in your professional life with death wishes expressed by patients?
   - o no
   - o yes if so,
     - ◊ professional
     - ◊ private

5. The didactic structure of the elective is well done.

strongly agree	rather agree	neither nor	rather disagree	strongly disagree
○	○	○	○	○

6. The structure of the elective in eLearning, self-study and (web) seminar is successful.

strongly agree	rather agree	neither nor	rather disagree	strongly disagree
○	○	○	○	○

7. The contents of the eLearning are presented in an understandable way.

strongly agree	rather agree	neither nor	rather disagree	strongly disagree
○	○	○	○	○

8. The lecturers appear competent.

strongly agree	rather agree	neither nor	rather disagree	strongly disagree
○	○	○	○	○

9. The accompanying (web) seminars make digitally conveyed content more understandable.

strongly agree	rather agree	neither nor	rather disagree	strongly disagree
○	○	○	○	○

10. The digital implementation of the web seminars is equivalent to a real encounter.

strongly agree	rather agree	neither nor	rather disagree	strongly disagree
○	○	○	○	○

11. The time frame for the exchange among each other is appropriate.

strongly agree	rather agree	neither nor	rather disagree	strongly disagree
○	○	○	○	○

12. The content of the elective prepares well for the simulated conversations.

strongly agree	rather agree	neither nor	rather disagree	strongly disagree
○	○	○	○	○

13. Through the simulated conversations, I have gained confidence in handling expressed desire to die.

strongly agree	rather agree	neither nor	rather disagree	strongly disagree
○	○	○	○	○

14. The elective has changed my attitude towards desire to die.

strongly agree	rather agree	neither nor	rather disagree	strongly disagree
○	○	○	○	○

15. Overall, I am satisfied with the elective course.

strongly agree	rather agree	neither nor	rather disagree	strongly disagree
○	○	○	○	○

16. I can imagine support a patient during voluntarily stopping eating and drinking (VSED).

strongly agree	rather agree	neither nor	rather disagree	strongly disagree
○	○	○	○	○

17. I can imagine offering physician assisted suicide to a patient.

strongly agree	rather agree	neither nor	rather disagree	strongly disagree
○	○	○	○	○

18. I can imagine carrying out death by request (forbidden in Germany).

strongly agree	rather agree	neither nor	rather disagree	strongly disagree
○	○	○	○	○

Please rate your knowledge / skills / abilities in relation to the following 13 statements!
Use the German school grading system from **1 = very good to 6 = insufficient**.
Take two points in time into consideration:
The <u>retrospective self-assessment before</u> starting the elective "Dealing with desires to die" and
the <u>current self-assessment after</u> completing the elective "Dealing with desires to die".

1. I believe that dealing with patients' desire to die is an important issue in my future professional work.

**retrospective**	
**current**	

2. I am aware of my own attitude to the issue of desire to die..

**retrospective**	
**current**	

3. I can reflect on the influence of norms and values on my attitude towards the desire to die.

**retrospective**	
**current**	

4. I have the confidence to proactively address a desire to die with patients.

**retrospective**	
**current**	

5. I can perceive and recognise a desire to die expressed by patients.

**retrospective**	
**current**	

6. I experience expressions of a desire to die by patients as burdensome.

**retrospective**	
**current**	

7. I recognise the different functions of a desire to die.

**retrospective**	
**current**	

8. I know about the meaning and reasons of a desire to die.

**retrospective**	
**current**	

9. I know the legal basis of assisted dying in Germany.

**retrospective**	
**current**	

10. I am able to define the different forms of assisted dying.

**retrospective**	
**current**	

11. I know my resilience resources.

**retrospective**	
**current**	

12. I know symptoms that belong to the final phase of dying.

**retrospective**	
**current**	

13. I trust myself to care for a patient with a desire to die.

**retrospective**	
**current**	
