# Supplementary material for: Handling the desire to die– evaluation of an elective course for medical students
Source: BMC Med Educ. 2024 Mar 18;24:279. doi: 10.1186/s12909-024-05269-6 (PMC10946106; doi:10.1186/s12909-024-05269-6)
Supplement: Supplementary file 2 — Supplementary Material 2 [file 12909_2024_5269_MOESM2_ESM.pdf]

Vignette

A patient aged 45 was diagnosed with breast cancer in 2018. In 2020, metastases were detected in the liver and bones and there was also exulcerating metastasis of the breast. The patient's general condition increasingly deteriorated and she suffered above all from severe pain and psychological stress due to the malignant tumour wound. The patient withdrew more and more and, during the time-consuming dressing changes, said that she simply couldn't and didn't want to go on.
Despite the introduction of palliative and symptom-relieving measures, the patient experiences hardly any relief from her suffering. She also consumes less and less food due to increasing gastrointestinal symptoms and says "I don't eat or drink any more and I don't want to be fed artificially!".

Analyse the example using the following categories:
1) Is the patient expressing a wish to hasten death? If so, how?
2) Which form of "assisted dying" corresponds to the patient's wish to hasten death? Decide on one of the following terms: "death on request", "renunciation of therapy", "acceptance of death", "assisted suicide". Explain the term.
3) What background and causes do you suspect are behind the expressed wish to hasten death?
4) What significance could the wish to die have for the patient?
5) How would you deal with this situation?
